# Retention Forces of Monolithic CAD/CAM Crowns Adhesively Cemented to Titanium Base Abutments—Effect of Saliva Contamination Followed by Cleaning of the Titanium Bond Surface

**DOI:** 10.3390/ma14123375

**Published:** 2021-06-18

**Authors:** Felix Burkhardt, João Pitta, Vincent Fehmer, Philippe Mojon, Irena Sailer

**Affiliations:** Division of Fixed Prosthodontics and Biomaterials, University Clinics of Dental Medicine, University of Geneva, 1211 Geneva, Switzerland; joao.pitta@unige.ch (J.P.); vincent.fehmer@unige.ch (V.F.); philippe.mojon@unige.ch (P.M.); irena.sailer@unige.ch (I.S.)

**Keywords:** bonding, CAD-CAM, implant dentistry, pull-off, retention force, saliva contamination, titanium base abutment

## Abstract

The aim of this study was to investigate the effects of saliva contamination and the cleaning of the bond surface of titanium base (ti-base) abutments on the bonding stability and retention force values. The bond surface of the ti-base abutments was treated with airborne-particle abrasion. After contamination, the ti-base abutments underwent different cleaning protocols: water spray (H_2_O); alcohol (ALC); suspension of zirconium particles (SZP); reapplied airborne-particle abrasion (APA); and a control condition without contamination and cleaning (CTR). All lithium disilicate crowns were bonded to the ti-base abutments using a primer and a self-curing composite. Bonded specimens underwent thermo-mechanical aging. Bond failure analysis and pull-off testing were performed. Bond failure occurred more frequently in groups H_2_O, ALC, SZP, and APA (*p* < 0.05). Significant differences in retention force values were only found between CTR and ALC (*p* < 0.05). Specimens which did not show bond failure after ageing had higher retention force values than the specimens that showed bond failure (*p* < 0.05). Saliva contamination with cleaning can degrade the bonding properties to titanium. For the retention force values, only the protocol with alcohol after contamination could not restore the values.

## 1. Introduction

Digitalization in the prosthetic implant dentistry, which started its development in dental laboratories, is now established in dental offices for the chairside fabrication of single-implant restorations [1]. To enable the fabrication and integration of full-contour all-ceramic restorations milled from monolithic ingots with a prefabricated connection, the titanium base (ti-base) abutment serves as a standardized connection [2,3]. The impression can be taken in a full-digital protocol using two different techniques: (1) at the implant level, with a scan body directly screwed on the implant [4]; (2) at the abutment level, with the ti-base abutment connected to the implant and a scan body clipped on the abutment [5].

Studies have shown that the adhesive cementation of the restoration to a ti-base abutment can be predictably achieved by applying an airborne-particle abrasion surface treatment to the titanium surface and conditioning the titanium and ceramic surface with a respective primer followed by a resin-based cement [6,7,8,9,10]. Primers are used to promote adhesion between dissimilar substrates. Although primers are substrate-specific, recent universal silane-based primers can be used with both ceramic and metal substrates since the mechanism of bonding is similar [11,12].

In general, a successful bonding is obtained in a controlled and clean situation extra-orally [13]. However, in some clinical situations contact with oral fluids such as saliva is barely avoidable, such as during digital impression-taking at the abutment level or during the chair-side try-in procedure of the restoration prior to bonding.

Saliva is a complex mixture that consists of a variety of electrolytes, small organic substances, proteins, peptides, and polynucleotides [14]. After the contamination of a bonding surface, non-covalent adsorptions of salivary proteins occur, resulting in an organic coating [15].

The effect of contamination on various bond surfaces has been thoroughly investigated with the rising use of adhesive bond systems utilized in the oral cavity environment [16,17]. Many studies have investigated the effect of contamination and possible cleaning methods for various ceramics, with a particular focus on zirconia, as it is a material often selected as an alternative to metal [18,19]. Studies have investigated the effect of the contamination of ceramic surfaces using thermal aging to simulate a certain period of use in the mouth [15,19,20,21]. Tensile or shear bond strength tests have been applied for most of these studies, while few have considered the geometric aspect of an implant abutment [22]. The geometric shape of an abutment and micro mechanical interlocking plays an additional role in retention besides bond forces and micro retentive forces [23].

Some of the studies on ceramics have shown that retreatment with airborne-particle abrasion and zirconium oxide particle suspension most predictably restores adhesive strength after the saliva contamination of zirconia, while cleaning with phosphoric acid or water did not reproduce predictable results [15,21]. It has been shown that the application of phosphoric acid to zirconia leaves a phosphor residue on the surface [24]. This might affect the bonding, since the adhesive bonds to zirconia and titanium are based on the chemical bond through functional monomers such as 10-methacryloyloxydecyl dihydrogen phosphate (MDP) [25,26,27]. In one study, the results of all cleaning protocols were equivalent to the control group and only the group cleaned with water exhibited lower retentive values [22], while in two other studies alcohol and water showed inferior results compared to a reapplied airborne-particle abrasion [20,28].

The cleaning possibilities of lithium disilicate ceramic after contamination with saliva were also investigated. One study compared cleaning with phosphoric acid, hydrofluoric acid, isopropanol, and an air-polishing device with sodium bicarbonate to clean contamination with saliva. Reapplied etching with hydrofluoric acid and phosphoric acid resulted in higher bonding values compared to the other cleaning methods [29]. Another study revealed that an alkaline suspension of zirconium oxide particles and 30% sodium silicate solution is more effective in decontaminating lithium disilicate ceramic than cleaning with water or hydrofluoric acid [30].

Even though the effect of saliva contamination with different cleaning protocols has been investigated for different ceramics, so far it has not been investigated how the contamination with saliva affects the adhesive properties to titanium and whether cleaning protocols are applicable and necessary.

Therefore, the aim of this study was to evaluate the influence of the saliva contamination of ti-base abutment bond surfaces on the retention forces of lithium disilicate crowns and to investigate the effect of different cleaning protocols.

The stated null hypotheses were: (1) saliva contamination and the cleaning of the ti-base surfaces do not influence the retention forces of lithium disilicate crowns on ti-base abutments; (2) different cleaning protocols of the contaminated ti-base surfaces do not influence the retention forces of lithium disilicate crowns on ti-base abutments.

## 2. Materials and Methods

### 2.1. Study Design and Setup

Sixty ti-base abutments (diameter 4.3 mm, GH 2.0 mm, CONELOG, Titanium base Crown, Camlog Biotechnologies AG, Basel, Switzerland) were divided into five groups (n = 12) according to the cleaning protocol after contamination with human saliva (Table 1). The sample size was based on previous studies using a similar methodology [8,9,10,31,32].

The design of the lithium disilicate crown (IPS e.maxCAD A3/A 16 (S), Ivoclar Vivadent, Schaan, Liechtenstein) was derived from an upper central incisor of 15 mm in dimensions and was modified by a horizontal ring, as used in previous studies, to provide a standardized pull-off testing procedure [9,10,32].

Sixty implants (diameter 4.3 mm GH 16 mm CONELOG, Camlog Biotechnologies AG, Basel, Switzerland) were fixed in a standardized way in acrylic resin blocks and bonded with self-curing acrylic (Technovit 4071, Kulzer GmbH, Hanau, Germany), according to ISO Norm 14801 (ISO, 2006), with a 3 mm exposed rough implant surface to simulate bone loss.

The ti-base abutments received a standardized airborne-particle abrasion surface treatment with 50 µm aluminum oxide at 2.0 bar of air pressure and were then cleaned in an ultrasonic alcohol bath for 5 min and dried with oil-free air.

### 2.2. Contamination

The human saliva was collected from a healthy male investigator. Prior to the collection procedure, the donor abstained from drinking and eating for 1.5 h. The contamination procedure was performed immediately after collection [29]. All the abutments except the ones from the control group (CTR) were inserted in saliva for 5 min. Immediately after contamination, all specimens were rinsed with water for 5 s. Subsequently, the abutments were divided among the study groups and cleaning was performed according to the respective protocol (Table 1).

### 2.3. Fabrication of Specimens

Lithium disilicate crowns were etched with 5% hydrofluoric acid (IPS Ceramic Etching Gel, Ivoclar Vivadent AG, Schaan, Liechtenstein) for 20 s. Subsequently, the crowns were cleaned in an ultrasonic bath with alcohol (Micro 10+, Unident SA, Geneva, Switzerland) for 4 min and dried with oil-free air. Adhesive cementation was performed according to the manufacturer’s recommendation using a primer which was left for 60 s on the surface (Monobond Plus, Ivoclar Vivadent, Schaan, Liechtenstein), followed by the use of an auto-polymerization composite (Multilink Hybrid Abutment HO0, Ivoclar Vivadent, Schaan, Liechtenstein). A slight excess of cement at the external margin was left to polymerization and covered with glycerin gel (Liquid Strip Glycerine Gel, Ivoclar Vivadent, Schaan, Liechtenstein). After complete polymerization, excess cement removal and the polishing of the margin were performed.

The restorations were fixed on the implants with a torque of 20 N cm using a torque wrench (Camlog Biotechnologies AG, Basel, Switzerland). The abutment screw access channels were closed with a polytetrafluoroethylene tape (Teflon, Chemours Co., Wilmington, NC, USA) and a light-polymerized composite resin (Tetric EvoCeram, Ivoclar Vivadent, Schaan, Liechtenstein).

### 2.4. Thermo-Mechanical Aging

All the specimens underwent aging through simultaneous thermocycling (temperature: 5 to 55 °C; dwell time: 120 s) and chewing simulation (cycles: 1,200,000; load: 49 N; frequency: 1.67 Hz) (Chewing Simulator CS-4.4, SD Mechatronik GmbH, Feldkirchen-Westerham, Germany) at a 30° angulation. A steatite sphere (∅ 6 mm) was used as an antagonist. This thermo-mechanical aging protocol has been reported to simulate five years of oral service [33].

### 2.5. Bond Failure Analysis

Specimens were examined to detect loss of retention or the presence of a naked-eye perceptible movement between the crown and ti-base abutment (macro-movement) after aging. If no such event was recorded, an evaluation under a light microscope (magnification 57×) (Olympus SZX9, Olympus, Tokyo, Japan) was conducted by two independent examiners (JP, FB) to control the presence of microscopic movements (micro-movements) between the crown and the ti-base abutment which are not perceptible nor visible by a naked-eye, as described in previous studies [9,10,32].

### 2.6. Pull-off Test

Retention force (N) was determined in a universal testing machine (Shimadzu AGS-X series, Shimadzu, Kyoto, Japan) with a 10 kN load cell at a crosshead speed of 0.5 mm/min. The horizontal ring of the crown was supported by an individualized holder assuring a constant pull-off direction in the insertion axis of the crown on the ti-base abutment. Maximum retention force was recorded using software (TRAPEZIUM X, V.1.4.4, Shimadzu, Kyoto, Japan) for each specimen and the perception of an acoustic click was recorded whenever it occurred (click = 1) or not (no click = 0) at the moment of debonding.

### 2.7. Scanning Electron Microscope Surface Analysis

For a surface analysis, one additional ti-base abutment for each group (n = 1) underwent contamination and cleaning. Five ti-base abutments were analyzed under a scanning electron microscope (SEM) (Zeiss Sigma 300 VP, Carl Zeiss Microscopy GmbH, Jena, Germany) for the surface characterization of the titanium surfaces after each cleaning protocol.

### 2.8. Statistical Analysis

The collected data were statistically analyzed with the SPSS software (IBM SPSS Statistics; IBM Corp, Armonk, NY, USA, v26) with a significance value set at α = 0.05. The Shapiro—Wilk test did not confirm a normal distribution; consequently, non-parametric Kruskal—Wallis tests were conducted to analyze the retention forces. To analyze the distribution of the observed bond failures and the acoustic click, a chi-squared test was used. The same test was used to evaluate the association between bond failure and acoustic click. The association between retention forces and bond failure as well as the perception of acoustic clicks was performed using a *t*-test.

## 3. Results

After aging, no loss of retention or macro-movements was detected. Micro-movements occurred more frequently with the cleaning protocols of H_2_O, ALC, SZP, or APA (*p* < 0.05).

**Figure 1:**
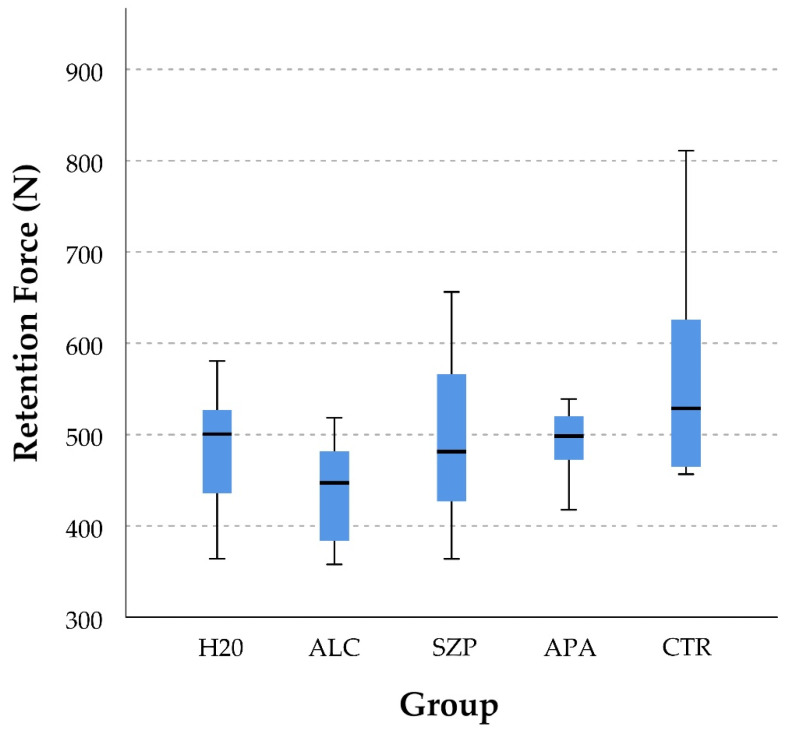
Boxplot of the median (IQR) retention force (N) among the five groups.

The retention forces varied from 439 N (median) in group ALC to 562 N (median) in group CTR (Figure 1), and significant differences were only found between these two groups (*p* < 0.05) (Table 2).

A significant association between bond failure and acoustic click was found (*p* < 0.05). Specimens that did not show micro-movements had higher mean retention force values (554 ± 26 N) than specimens that revealed micro-movements (473 ± 10 N) (*p* < 0.05). Specimens with an acoustic click at the moment of debonding revealed higher bond values (546 ± 30 N) than specimens without a click (478 ± 9 N) (*p* < 0.05).

**Figure 2:**
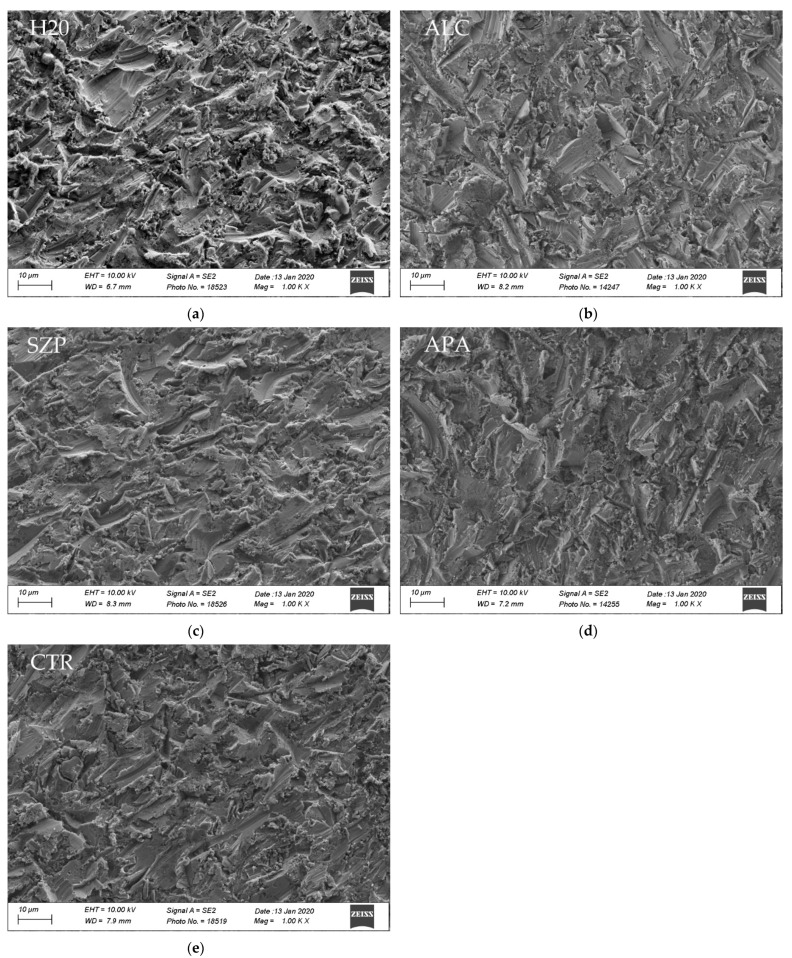
SEM photographs of the titanium bond surface of each group after cleaning (1000×): (**a**) water (H_2_O); (**b**) ultrasonic alcohol bath (ALC); (**c**) alkaline suspension of zirconium oxide particles (SZP); (**d**) reapplied standardized airborne-particle abrasion (APA); (**e**) control without contamination (CTR).

The SEM surface analysis did not reveal visually recognizable differences (Figure 2a–e).

## 4. Discussion

The present study showed that the retention forces of lithium disilicate crowns on ti-base abutments were significantly influenced by saliva contamination and the cleaning of the titanium surface; however, no differences were found between the different cleaning protocols that were applied after contamination. Therefore, the first null hypothesis was rejected, but the second one could not be rejected.

Although saliva contamination followed by a cleaning procedure revealed a trend of decreased retention forces compared with the non-contamination condition, a significant reduction could only be found for contaminated surfaces cleaned with alcohol. On the one hand, the saliva contamination may negatively affect the adhesion, as reported in previous investigations [15,20,21,22,28,29]. Nevertheless, the different cleaning procedures, all except alcohol, appear to partially restore the retention forces to values close to that of the control group.

The decreased retention forces obtained in the group cleaned with alcohol are in line with the results of other studies on the decontamination of different ceramics [20,28,29]. Even though ultrasonic cleaning in alcohol has been shown to be an important factor in improving adhesion to zirconia ceramics after airborne-particle abrasion [34], the reduced cleaning potential of alcohol after contamination might be explained by the fact that alcohol may fix proteins to surfaces and does not help to remove them [35]. Regarding the other cleaning protocols, the use of a suspension of zirconium oxide particles was confirmed as a valid option for decontaminating the titanium surfaces, as has been shown as well for ceramics [15,21,22]. This surface cleaning method is described to remove saliva phosphate impurities and improves adhesion by providing a clean bond surface [36]. Even though this protocol demonstrated no significantly different retention forces to water or re-applied airborne-particle abrasion, it appears to provide an improved bonding interface with fewer bonding failures and more events of perceived acoustic clicks than the other cleaning protocols. The remaining cleaning protocols showed more bonding failures (micro-movements), and fewer perceived acoustic clicks were detectable at the moment of debonding. Micro-movements observed after aging have been suggested as an initial sign of incipient bonding failure [9,10,32]. It was shown in the current study that microscopically detected micro-movements and the no perception of an acoustic click were a predictor of reduced retention force values. This finding on micro-movements is consistent with the results of other studies in which micro-movements were associated with reduced retention force values and described as an initial failure of the adhesive bond [3,9,10].

The SEM surface analysis did not reveal any visible remnants on the titanium sur-face either from saliva or cleaning residue. The findings are in line with a study conducted on porcelain veneers which also did not find any evidence of these remnants [37]. Another study detected minor remnants of zirconium oxide particles on ceramic surfaces [38]. For the more detailed visibility of the complete surface structure, the abutments should not have been uniquely dried before the SEM surface analysis but could also have been coated for the more accurate resolution of all structures present on the surface [39].

Reapplied airborne-particle abrasion following saliva contamination achieved retention force values which were reduced but not significantly different to those achieved in a non-contaminated condition in the control group, which is consistent with a study on zirconia [28]. However, due to the increased events of bonding failures and no perception of acoustic clicks, it was shown that the bonding interface does not correspond to a clinically acceptable outcome with the opened margin, which was visible under the microscope. At the same time, micro-movements were detected, which is a niche for the colonization of bacteria [40].

The results for the retention force values of the water cleaning protocol are not consistent with those of other studies on ceramics, where water led to significantly lower retention forces [21,22,28]. An explanation for this could be that the conducted protocol in our study consisted of active rinsing with a water spray for 1 min by a multifunctional syringe, which resulted in increased kinetic energy on the surface and might have favored the cleaning of the contamination [41].

The strengths of the present study are the dual aging protocol with thermal and mechanical chewing simulation, the high standardization of the airborne-particle abrasion of the ti-base abutments, and the use of a clinically used ti-base abutment and the pull-off testing setup, which allowed a consistent pull-off direction for all specimens. One limitation of this study is the in vitro protocol of contamination with saliva, which might be more complex in the oral cavity due to the presence of blood and contact contamination with oral soft tissue and may vary from patient to patient. Moreover, no group with only saliva contamination was integrated and the ti-base abutments were analyzed in the SEM without coating, which could have revealed more precise information. Additionally, X-ray photoelectron spectroscopy for chemical surface analysis might have revealed more precise information on the chemical composition of the bond surface after contamination and cleaning [42].

## 5. Conclusions

Within the limitations of this in vitro study, the following conclusions are drawn:

- contamination followed by cleaning can have a negative influence on the bonding properties to titanium and specific cleaning protocols performed better than others;
- regarding retention force, every protocol allowed the restoration of the bonding forces except for the protocol using alcohol.

## Figures and Tables

**Table 1:** Group identification and respective cleaning protocol applied.

Groups		Pre-Treatment	Contamination	Cleaning Protocol
Water	H_2_O	50 µm aluminum oxide by 2.0 bar of air pressure for 10 s/ ultrasonic alcohol bath for 5 min/dried with oil-free air	inserted in human saliva (5 min)	water-spray (1 min)
Alcohol	ALC			ultrasonic alcohol bath (5 min)
Suspension of zirconium particles	SZP			alkaline suspension of zirconium oxide particles (20 s)
Airborne-particle abrasion	APA			reapplied standardized airborne-particle abrasion
Control	CTR		Not applied	

**Table 2:** Bonding failures in absolute numbers; means with standard deviations (SD) and medians with interquartile ranges (IQR) of retention force values (N); statistical differences (*p* < 0.05) are indicated by different lowercase letters; means of noticeable acoustic click events at the moment of debonding (value = 1) and no click (value = 0).

Group	Bonding Failure				Retention Force		Click at Debonding
	No Failure	Micro- Movement	Macro- Movement	Loss of Retention	Mean (± SD)	Median (IQR)	
H_2_O	0	12	0	0	487 N (± 18.1)	501 N (101) ^ab^	0.08 (8%)
ALC	0	12	0	0	439 N (± 16.1)	447 N (107) ^b^	0.00 (0%)
SZP	6	6	0	0	501 N (± 26.4)	481 N (157) ^ab^	0.67 (67%)
APA	1	11	0	0	493 N (± 10.2)	498 N (59) ^ab^	0.00 (0%)
CTR	9	3	0	0	562 N (± 33.1)	529 N (187) ^a^	0.67 (67%)

## Data Availability

The data are contained in the article.
